# Approximations to the expectations and variances of ratios of tree properties under the coalescent

**DOI:** 10.1093/g3journal/jkac205

**Published:** 2022-08-11

**Authors:** Egor Lappo, Noah A Rosenberg

**Affiliations:** Department of Biology, Stanford University, Stanford, CA 94305, USA; Department of Biology, Stanford University, Stanford, CA 94305, USA

**Keywords:** coalescent theory, external branches, internal branches, time to the most recent common ancestor

## Abstract

Properties of gene genealogies such as tree height (*H*), total branch length (*L*), total lengths of external (*E*) and internal (*I*) branches, mean length of basal branches (*B*), and the underlying coalescence times (*T*) can be used to study population-genetic processes and to develop statistical tests of population-genetic models. Uses of tree features in statistical tests often rely on predictions that depend on pairwise relationships among such features. For genealogies under the coalescent, we provide exact expressions for Taylor approximations to expected values and variances of ratios $X_{n}/Y_{n}$, for all 15 pairs among the variables $\left\{H_{n},L_{n},E_{n},I_{n},B_{n},T_{k}\right\}$, considering *n* leaves and 2≤k≤n. For expected values of the ratios, the approximations match closely with empirical simulation-based values. The approximations to the variances are not as accurate, but they generally match simulations in their trends as *n* increases. Although *E_n_* has expectation 2 and *H_n_* has expectation 2 in the limit as n→∞, the approximation to the limiting expectation for $E_{n}/H_{n}$ is not 1, instead equaling $\pi^{2}/3-2\approx 1.28987$. The new approximations augment fundamental results in coalescent theory on the shapes of genealogical trees.

## Introduction

Coalescent theory models random genealogies conditional on assumptions about the evolutionary process (Hein *et al.* 2005; Wakeley 2009). In coalescent theory, a gene genealogy is a tree or network structure that represents a random draw from a coalescent model.

Genealogies in coalescent theory can be summarized using a variety of quantities. For example, for random tree-like genealogies with *n* lineages, the tree height *H_n_* records the sum of branch lengths on a path from a leaf to the root, and the tree length *L_n_* sums all branch lengths in the tree. The total length *E_n_* of external branches sums over leaves the lengths of paths from leaves to their nearest internal nodes, and the total length of internal branches, $I_{n}=L_{n}-E_{n}$, sums the lengths of all remaining branches.

Studies in coalescent theory have often investigated the properties of tree summaries conditional on assumptions of coalescent models, with the goal of understanding how shapes of the genealogies relate to processes such as population growth and migration (e.g. Slatkin 1996; Rosenberg and Feldman 2002). Because mutations can be viewed as occurring conditionally on underlying genealogies (Hudson 1990), features of genealogical shape affect the patterns of genetic variation produced by coalescent models that permit mutation. Thus, the understanding of summaries of tree shape predicted by coalescent models is a component of the interpretation of patterns of genetic variation in relation to evolutionary processes.

Initial results concerning summaries of genealogical shape focused on single quantities, producing results on quantities such as *H_n_* and *L_n_* (Kingman 1982; Hudson 1983, 1990; Tajima 1983). Studies soon examined the information that resides in the relationships between pairs of summaries; genetic variation statistics such as those of Tajima (1989) and Fu and Li (1993) can be viewed as assessing whether or not one aspect of a tree contains long branches in relation to another.

Recently, Arbisser *et al.* (2018) performed a detailed investigation of the relationship between *H_n_* and *L_n_* under coalescent models. They studied the mathematical relationship between these two quantities, computing under a standard coalescent model with a constant-sized population the covariance and correlation coefficient of *H_n_* and *L_n_*. Extending the work of Arbisser *et al.* (2018) on *H_n_* and *L_n_*, we (Alimpiev and Rosenberg 2022) reported covariances and correlations for all pairs of variables among $\left\{H_{n},L_{n},E_{n},I_{n},B_{n},T_{k}\right\}$, where *B_n_* is the mean of the lengths of the two basal branches of a genealogy and *T_k_* is the coalescence time from *k* to *k—*1 lineages, 2≤k≤n. Our compendium in Tables 1 and 2 of Alimpiev and Rosenberg (2022) summarizes pairwise relationships for several of the most commonly used features of coalescent tree shape, recording both new and previously known results.

**Table 1.** Definitions of random variables associated with various tree summaries.

Variable	Definition
*H_n_*	$\sum_{k=2}^{n}T_{k}$
*L_n_*	$\sum_{k=2}^{n}kT_{k}$
*E_n_*	$\sum_{i=1}^{n}e_{i}^{\left(n\right)}$
*I_n_*	$L_{n}-E_{n}$
*B_n_*	$\frac{1}{2}T_{2}+[\sum_{j=3}^{n-1}\sum_{k=2}^{j}\frac{1}{j(j-1)}T_{k}]+(\sum_{k=2}^{n}\frac{1}{n-1}T_{k})$

Here, *T_k_* is the random variable representing the coalescence time from *k* to *k—*1 lineages, and $e_{i}^{\left(n\right)}$ is the (random) length of the *i*th external branch of a tree with *n* leaves. We define *H_n_*, *L_n_*, and *E_n_* for n≥2, *I_n_* for n≥3, and *B_n_* for n≥4. The expression for *B_n_* follows a form that incorporates terms associated with all of its contributing branches, following p. 1400 of Uyenoyama (1997) and Section 2.6 of Alimpiev and Rosenberg (2022), and it can be simplified to $B_{n}=\sum_{k=2}^{n}\frac{1}{k-1}T_{k}$.

**Table 2.** Expectations and variances of properties of tree branch lengths.

*X_n_*	$E\left[X_{n}\right]$	$\underset{n\to \infty }{\lim}E\left[X_{n}\right]$	$\text{Var}\left[X_{n}\right]$	$\underset{n\to \infty }{\lim}\text{Var}\left[X_{n}\right]$
*H_n_*	$\frac{2(n-1)}{n}$	2	$8(S_{2,n}-1)-4{\left(\frac{n-1}{n}\right)}^{2}$	$\frac{4\pi^{2}}{3}-12\approx 1.15947$
*L_n_*	$2S_{1,n-1}$	∞	$4S_{2,n-1}$	$\frac{2\pi^{2}}{3}\approx 6.57974$
*E_n_*	2	2	$\{\begin{array}{cc} 4,n=2,\\ \frac{8}{\left(n-1)(n-2\right)}[S_{1,n-1}n-2(n-1)], & n>2. \end{array}$	0
*I_n_*	$2S_{1,n-1}-2$	∞	$4\left[\frac{2[S_{1,n-1}n-2(n-1)]}{\left(n-1)(n-2\right)}-\frac{2S_{1,n-1}}{n-1}+S_{2,n-1}\right]$	$\frac{2\pi^{2}}{3}\approx 6.57974$
*B_n_*	$2S_{2,n-1}-2+\frac{2}{n}$	$\frac{\pi^{2}}{3}-2\approx 1.28987$	$\frac{2(3S_{2,n-1}n^{2}-2S_{2,n-1}^{2}n^{2}+n^{2}-4S_{2,n-1}n+3n-4)}{n^{2}}$	$-\frac{\pi^{4}}{9}+\pi^{2}+2\approx 1.04637$
*T_k_*	$\frac{2}{k(k-1)}$	$\frac{2}{k(k-1)}$	$\frac{4}{k^{2}{\left(k-1\right)}^{2}}$	$\frac{4}{k^{2}{\left(k-1\right)}^{2}}$

These expressions can be found in Alimpiev and Rosenberg (2022). Note that for *L_n_* and *I_n_*, although the limiting variance is finite, the expectation is infinite (Tavaré *et al.* 1997; Wakeley 2009, p. 76).

In addition to computing the covariance and correlation coefficient of *H_n_* and *L_n_*, Arbisser *et al.* (2018) also found approximations to the expectation and variance of the ratio $H_{n}/L_{n}$ under the coalescent model. This ratio gives a summary of the joint distribution of *H_n_* and *L_n_* that characterizes the relative magnitudes of the variables—a feature not captured by their covariance or correlation. Arbisser *et al.* (2018) found that although the approximation to $\text{Var}[H_{n}/L_{n}]$ differed noticeably from the exact value, as obtained by numerical integration and simulations of the coalescent model, the approximation to $E[H_{n}/L_{n}]$ was quite accurate.

In this article, we extend the work of Arbisser *et al.* (2018) to compute approximations to the expectations and variances for ratios of the 14 remaining pairs among $\left\{H_{n},L_{n},E_{n},I_{n},B_{n},T_{k}\right\}$. The study performs for the expectation and variance of coalescent ratios an analogous extension of Arbisser *et al.* (2018) to that performed by Alimpiev and Rosenberg (2022) for the covariance and correlation coefficient.

## Materials and methods

### Tree variables

We work with a haploid population of constant size *N* that follows a standard coalescent model. Time is measured in units of *N* generations. In this section, we recall the definitions of the coalescence time *T_k_* and tree properties *H_n_*, *L_n_*, *E_n_*, *I_n_*, and *B_n_* for sample size n≥2 and 2≤k≤n.

*T_k_* is defined to be a random variable representing the time to coalescence of *k* to *k—*1 lineages, for 2≤k≤n. Variable *T_k_* has exponential probability density function

$$f_{T_{k}}(t)=\left(\begin{array}{c} k\\ 2 \end{array}\right)e^{-\left(\begin{array}{c} k\\ 2 \end{array}\right)t}.$$

The expectation and variance of *T_k_* are

(1) $$E\left[T_{k}\right]=\frac{2}{k(k-1)},$$

(2) $$\text{Var}\left[T_{k}\right]=\frac{4}{k^{2}{\left(k-1\right)}^{2}}.$$

**Fig. 1.**
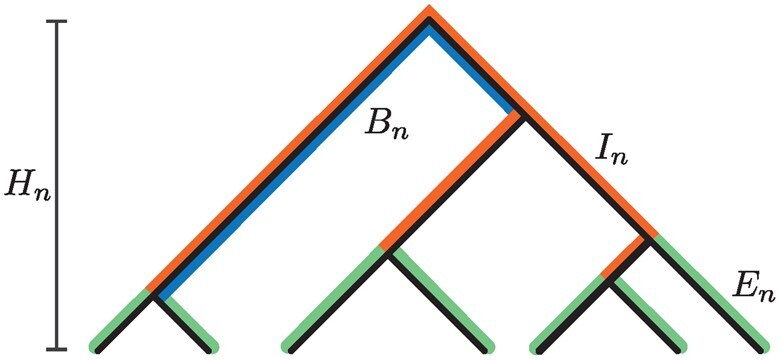
Properties of genealogical trees. The tree height is *H_n_*. The sum of the lengths of all branches is *L_n_*. External branches have total length *E_n_* (green). Internal branches have total length *I_n_* (orange). Basal branches have mean length *B_n_* (blue).

The tree properties *H_n_*, *L_n_*, *E_n_*, *I_n_*, and *B_n_* are defined in terms of the *T_k_*. Visual depictions of these properties appear in Fig. 1, and mathematical definitions of these quantities appear in Table 1.

We define $S_{p,n}=\sum_{k=1}^{n}k^{-p}$ as a useful shorthand. The limit $\underset{n\to \infty }{\lim}S_{p,n}=S_{p,\infty }$ is the Riemann zeta function, usually denoted ζ(p). In particular, $S_{1,\infty }$ diverges, $S_{2,\infty }=\pi^{2}/6\approx 1.64493$, and $S_{3,\infty }$ is Apéry’s constant, approximately 1.20206.

### Taylor approximations to expectations and variances of ratios

To compute approximate expressions for expected values and variances of the ratios of various tree properties, we rely on Taylor approximations. In particular, consider random variables *X* and *Y* with E[X],E[Y]≠0. For the expectation, we have (second-order) approximation (Elandt-Johnson and Johnson 1999, eq. 3.88):

(3) $$E\left[\frac{X}{Y}\right]\approx \frac{E\left[X\right]}{E\left[Y\right]}-\frac{\text{Cov}[X,Y]}{E{\left[Y\right]}^{2}}+\frac{E\left[X\right]\text{Var}\left[Y\right]}{E{\left[Y\right]}^{3}}.$$

For the variance, we have (first-order) approximation (Stuart and Ord 1994, eq. 10.17):

(4) $$\text{Var}\left[\frac{X}{Y}\right]\approx {\left(\frac{E\left[X\right]}{E\left[Y\right]}\right)}^{2}\left(\frac{\text{Var}\left[X\right]}{E{\left[X\right]}^{2}}-\frac{2\text{Cov}[X,Y]}{E\left[X\right]E\left[Y\right]}+\frac{\text{Var}\left[Y\right]}{E{\left[Y\right]}^{2}}\right).$$

We use $\tilde{E}\left[X/Y\right]$ and $\tilde{\text{Var}}\left[X/Y\right]$ to denote approximations from equations (3) and (4). For both the expectation and the variance, we also take the n→∞ limit of the approximations.

### Exact expectations, variances, and covariances of tree properties

Expected values and variances of variables *H_n_*, *L_n_*, *E_n_*, *I_n_*, *B_n_*, and *T_k_* that are used in equations (3) and (4) are known, in many cases, from early studies in coalescent theory (Fu and Li 1993; Tavaré *et al.* 1997; Wakeley 2009). We summarize these expectations and variances in Table 2.

The covariances compiled by Alimpiev and Rosenberg (2022) appear in Table 3. In the case of pairs (*E_n_*, *B_n_*) and (*I_n_*, *B_n_*), the covariances are approximate, as described by Alimpiev and Rosenberg (2022).

**Table 3.** Covariances of pairs of variables that summarize genealogical trees.

(*X_n_*, *Y_n_*)	$\text{Cov}[X_{n},Y_{n}]$	$\underset{n\to \infty }{\lim}\text{Cov}[X_{n},Y_{n}]$
*H_n_*, *T_k_*	$\frac{4}{k^{2}{\left(k-1\right)}^{2}}$	$\frac{4}{k^{2}{\left(k-1\right)}^{2}}$
*H_n_*, *L_n_*	$4S_{2,n-1}-4+\frac{4}{n}$	$\frac{2\pi^{2}}{3}-4\approx 2.57974$
*H_n_*, *E_n_*	$\frac{4}{n}$	0
*H_n_*, *I_n_*	$4S_{2,n-1}-4$	$\frac{2\pi^{2}}{3}-4\approx 2.57974$
*H_n_*, *B_n_*	$\frac{4[S_{3,n-1}n^{2}-3S_{2,n-1}n^{2}+(4n+1)(n-1)]}{n^{2}}$	$-2\pi^{2}+4\zeta (3)+16\approx 1.06902$
*L_n_*, *T_k_*	$\frac{4}{k{\left(k-1\right)}^{2}}$	$\frac{4}{k{\left(k-1\right)}^{2}}$
*L_n_*, *E_n_*	$\frac{4S_{1,n-1}}{n-1}$	0
*L_n_*, *I_n_*	$4S_{2,n-1}-\frac{4S_{1,n-1}}{n-1}$	$\frac{2\pi^{2}}{3}\approx 6.57974$
*L_n_*, *B_n_*	$\frac{4[S_{3,n-1}n-S_{2,n-1}n+n-1]}{n}$	$-\frac{2\pi^{2}}{3}+4\zeta (3)+4\approx 2.22849$
*E_n_*, *T_k_*	$\frac{4}{k(k-1)(n-1)}$	0
*E_n_*, *I_n_*	$\frac{4S_{1,n-1}}{n-1}-\frac{8S_{1,n-1}n}{\left(n-1)(n-2\right)}+\frac{16}{n-2}$	0
*E_n_*, *B_n_*	$\frac{4(S_{2,n-1}n-n+1)}{n(n-1)}$	0
*I_n_*, *T_k_*	$\frac{4(n-k)}{k{\left(k-1\right)}^{2}(n-1)}$	$\frac{4}{k{\left(k-1\right)}^{2}}$
*I_n_*, *B_n_*	$\frac{4(S_{3,n-1}n-S_{2,n-1}n+n-S_{3,n-1}-1)}{n-1}$	$-\frac{2\pi^{2}}{3}+4\zeta (3)+4\approx 2.22849$
*B_n_*, *T_k_*	$\frac{4}{k^{2}{\left(k-1\right)}^{3}}$	$\frac{4}{k^{2}{\left(k-1\right)}^{3}}$

For pairs involving *E_n_* or *I_n_*, expressions apply for n≥3; expressions involving *B_n_* apply for n≥4. The expressions can be found in Alimpiev and Rosenberg (2022).

### Evaluating the approximations

**Fig. 2.**
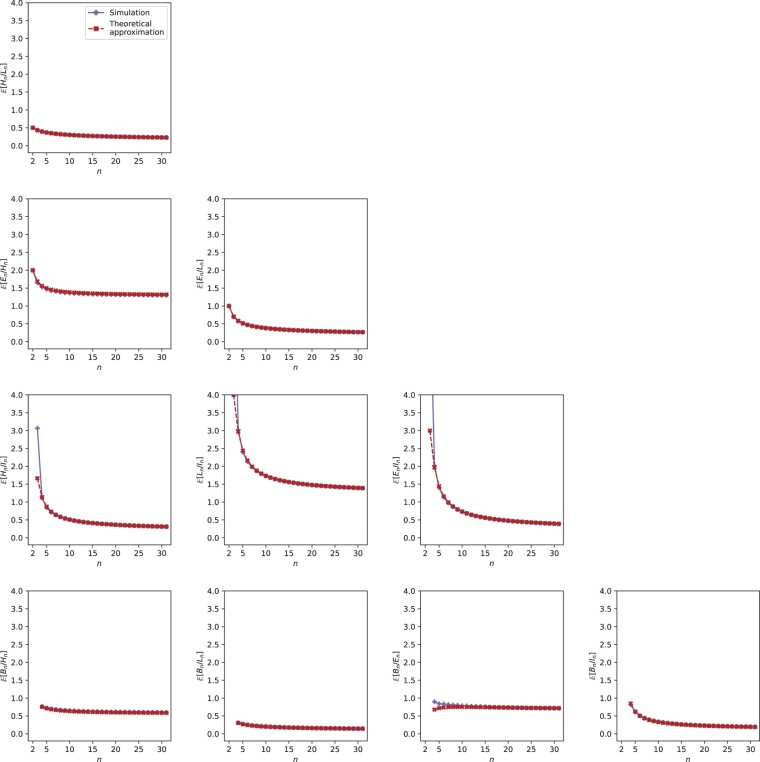
Simulated and theoretical approximations of expectations of ratios of pairs of variables, plotted as functions of sample size *n*. Expressions for theoretical values are taken from Table 4.

**Fig. 3.**
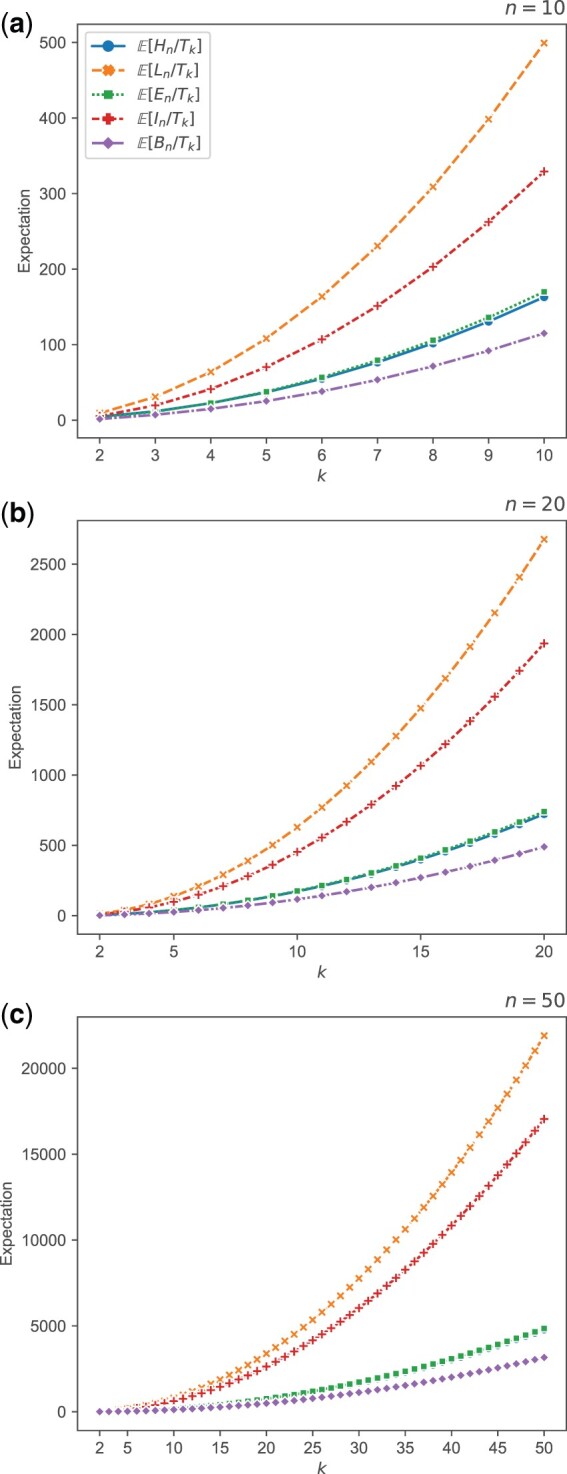
Theoretical approximations $\tilde{E}\left[X/T_{k}\right]$ for variables *X* in $\left\{H_{n},L_{n},E_{n},I_{n},B_{n}\right\}$, plotted as functions of *k* for *n *=* *10, *n *=* *20, and *n *=* *50. The expressions plotted are taken from Table 4.

**Fig. 4.**
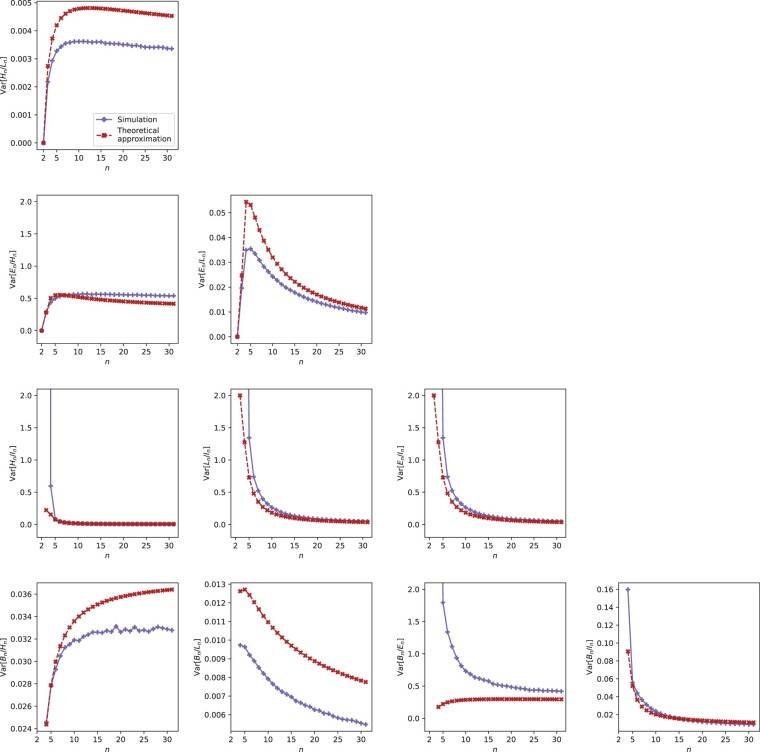
Simulated and theoretical approximations of variances of ratios of pairs of variables, plotted as functions of sample size *n*. Expressions for theoretical values are taken from Table 5.

**Fig. 5.**
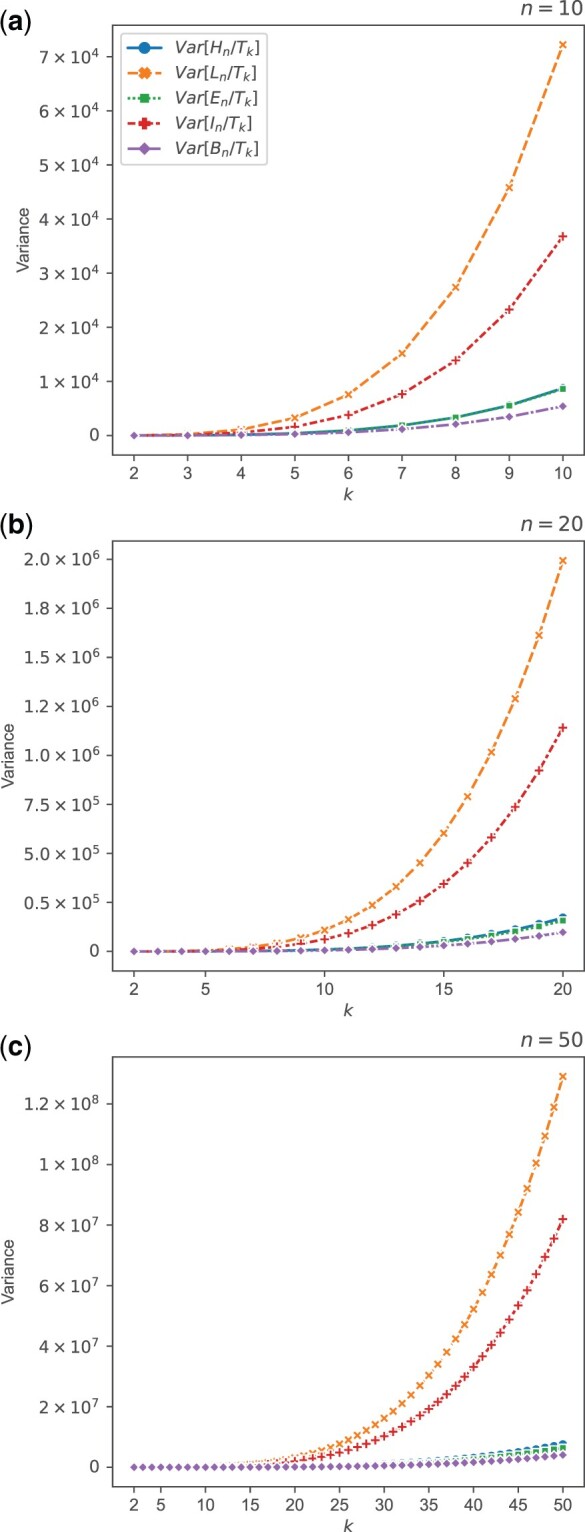
Theoretical approximations $\tilde{\text{Var}}\left[X/T_{k}\right]$ for variables *X* in $\left\{H_{n},L_{n},E_{n},I_{n},B_{n}\right\}$, plotted as functions of *k* for *n *=* *10, *n *=* *20, and *n *=* *50. The expressions plotted are taken from Table 5.

For each of 15 pairs of random variables, considering *H_n_*, *L_n_*, *E_n_*, *I_n_*, and *B_n_* as well as *T_k_*, we substitute expressions from Tables 2 and 3 into equations (3) and (4) to obtain approximate expectations and variances for ratios of pairs of variables. For each pair, we choose one variable for the numerator and the other for the denominator; approximate expectations and variances for the reciprocals can be obtained similarly. We present the approximations in Tables 4 and 5, and we plot them in Figs. 2–5.

**Table 4.** Approximations to expectations of ratios of pairs of variables.

(*X_n_*, *Y_n_*)	$\tilde{E}\left[X_{n}/Y_{n}\right]$	$\underset{n\to \infty }{\lim}\tilde{E}\left[X_{n}/Y_{n}\right]$
*H_n_*, *T_k_*	$\frac{\left(2k^{2}-2k-1)n-2k(k-1\right)}{n}$	$2k^{2}-2k-1$
*H_n_*, *L_n_*	$\frac{n-1}{S_{1,n-1}n}-\frac{S_{2,n-1}n-n+1}{S_{1,n-1}^{2}n}+\frac{S_{2,n-1}(n-1)}{S_{1,n-1}^{3}n}$	0
*E_n_*, *H_n_*	$\frac{n(2S_{2,n}n^{2}-2n^{2}-n+1)}{{\left(n-1\right)}^{3}}$	$\frac{\pi^{2}}{3}-2\approx 1.28987$
*H_n_*, *I_n_*	$\frac{n-1}{(S_{1,n-1}-1)n}-\frac{S_{2,n-1}-1}{{\left(S_{1,n-1}-1\right)}^{2}}+\frac{S_{2,n-1}(n-1)(n-2)-4n+4S_{1,n-1}+4}{{\left(S_{1,n-1}-1\right)}^{3}n(n-2)}$	0
*B_n_*, *H_n_*	$\frac{S_{2,n-1}n-n+1}{n-1}+\frac{3S_{2,n-1}n^{2}-S_{3,n-1}n^{2}-4n^{2}+3n+1}{{\left(n-1\right)}^{2}}+\frac{\left(S_{2,n-1}n-n+1)(2S_{2,n}n^{2}-3n^{2}+2n-1\right)}{{\left(n-1\right)}^{3}}$	$\frac{\pi^{4}}{18}-\frac{\pi^{2}}{6}-\zeta (3)-2\approx 0.56463$
*L_n_*, *T_k_*	$2S_{1,n-1}k^{2}-(2S_{1,n-1}+1)k$	∞
*E_n_*, *L_n_*	$\frac{(S_{1,n-1}^{2}+S_{2,n-1})n-2S_{1,n-1}^{2}-S_{2,n-1}}{S_{1,n-1}^{3}(n-1)}$	0
*L_n_*, *I_n_*	$\frac{\left(S_{1,n-1}^{3}+S_{2,n-1})(n-1)(n-2)-S_{1,n-1}^{2}(2n^{2}-7n+2)+S_{1,n-1}(n^{2}-8n+8\right)}{{\left(S_{1,n-1}-1\right)}^{3}(n-1)(n-2)}$	1
*B_n_*, *L_n_*	$\frac{S_{2,n-1}n-n+1}{S_{1,n-1}n}+\frac{S_{2,n-1}n-S_{3,n-1}n-n+1}{S_{1,n-1}^{2}n}+\frac{S_{2,n-1}(S_{2,n-1}n-n+1)}{S_{1,n-1}^{3}n}$	0
*E_n_*, *T_k_*	$\frac{k(k-1)(2n-3)}{n-1}$	2k(k−1)
*E_n_*, *I_n_*	$\frac{S_{1,n-1}^{2}(n^{2}-2n+4)-S_{1,n-1}(2n^{2}-n-2)+(S_{2,n-1}+1)(n-1)(n-2)}{{\left(S_{1,n-1}-1\right)}^{3}(n-1)(n-2)}$	0
*B_n_*, *E_n_*	$\frac{\left(n^{2}+2S_{1,n-1}n-8n+8)(S_{2,n-1}n-n+1\right)}{n(n-1)(n-2)}$	$\frac{\pi^{2}}{6}-1\approx 0.64493$
*I_n_*, *T_k_*	$2k(k-1)(S_{1,n-1}-1)-\frac{k(n-k)}{n-1}$	∞
*B_n_*, *I_n_*	$\frac{S_{2,n-1}n-n+1}{(S_{1,n-1}-1)n}+\frac{\left(S_{2,n-1}n-n+1)[S_{2,n-1}(n-1)(n-2)-4n+4S_{1,n-1}+4\right]}{{\left(S_{1,n-1}-1\right)}^{3}n(n-1)(n-2)}+\frac{S_{2,n-1}n-(S_{3,n-1}+1)(n-1)}{{\left(S_{1,n-1}-1\right)}^{2}(n-1)}$	0
*B_n_*, *T_k_*	$\frac{2k(k-1)(S_{2,n-1}n-n+1)}{n}-\frac{1}{k-1}$	$\frac{1}{3}(\pi^{2}-6)k(k-1)-\frac{1}{k-1}$

Expressions involving *E_n_* or *I_n_* apply for n≥3; expressions involving *B_n_* apply for n≥4. The value for (*H_n_*, *L_n_*) follows equation 15 of Arbisser *et al.* (2018). The expressions are obtained using equation 3 and Tables 2 and 3.

**Table 5.** Approximations to variances of ratios of pairs of variables.

(*X_n_*, *Y_n_*)	$\tilde{\text{Var}}\left[X_{n}/Y_{n}\right]$	$\underset{n\to \infty }{\lim}\tilde{\text{Var}}\left[X_{n}/Y_{n}\right]$
*H_n_*, *T_k_*	$\frac{2k(k-1)[k(k-1)S_{2,n}n-(k^{2}-k+1)n+1]}{n}$	$\frac{1}{3}k(k-1)[(\pi^{2}-6)k^{2}-(\pi^{2}-6)k-6]$
*H_n_*, *L_n_*	${\left(\frac{n-1}{S_{1,n-1}n}\right)}^{2}\left[\frac{2(S_{2,n}-1)n^{2}-{\left(n-1\right)}^{2}}{{\left(n-1\right)}^{2}}-\frac{2[S_{2,n-1}n-(n-1)]}{S_{1,n-1}(n-1)}+\frac{S_{2,n-1}}{S_{1,n-1}^{2}}\right]$	0
*E_n_*, *H_n_*	$\frac{[2S_{1,n-1}n(n-1)+2S_{2,n}n^{2}(n-2)-(n^{2}-3)(3n-2)]n^{2}}{{\left(n-1\right)}^{4}(n-2)}$	$\frac{\pi^{2}}{3}-3\approx 0.28987$
*H_n_*, *I_n_*	$\frac{2S_{2,n}n^{2}-3n^{2}+2n-1}{{\left(S_{1,n-1}-1\right)}^{2}n^{2}}+\frac{1}{{\left(S_{1,n-1}-1\right)}^{4}}\left[\frac{\left[[S_{2,n-1}(n-2)-4](n-1)+4S_{1,n-1}](n-1\right)}{n^{2}(n-2)}-\frac{2(S_{1,n-1}-1)(S_{2,n-1}-1)(n-1)}{n}\right]$	0
*B_n_*, *H_n_*	$\frac{(4S_{3,n-1}n^{2}+4S_{2,n}n^{2}+11n^{2}-5n-10){\left(n-1\right)}^{2}-S_{2,n-1}(4S_{3,n-1}n^{2}+8S_{2,n}n^{2}+13n^{2}-9n-12)n(n-1)+4S_{2,n-1}^{2}(S_{2,n}n^{2}+n^{2}-n-1)n^{2}}{2{\left(n-1\right)}^{4}}$	$\frac{\pi^{6}}{108}-\frac{\pi^{4}}{18}-\frac{3\pi^{2}}{4}+\frac{11}{2}+2\zeta (3)-\frac{\pi^{2}\zeta (3)}{3}\approx 0.03744$
*L_n_*, *T_k_*	$k^{2}{\left(k-1\right)}^{2}S_{1,n-1}^{2}\left[\frac{S_{2,n-1}}{S_{1,n-1}^{2}}-\frac{2}{(k-1)S_{1,n-1}}+1\right]$	∞
*E_n_*, *L_n_*	$\frac{2S_{1,n-1}^{3}n-S_{1,n-1}^{2}(6n-8)+S_{2,n-1}(n-1)(n-2)}{S_{1,n-1}^{4}(n-1)(n-2)}$	0
*L_n_*, *I_n_*	$\frac{2S_{1,n-1}^{3}n-S_{1,n-1}^{2}(6n-8)+S_{2,n-1}(n-1)(n-2)}{{\left(S_{1,n-1}-1\right)}^{4}(n-1)(n-2)}$	0
*B_n_*, *L_n_*	$\frac{S_{1,n-1}^{2}[-2S_{2,n-1}^{2}n^{2}+S_{2,n-1}(3n-4)n+n^{2}+3n-4]+4S_{1,n-1}(S_{2,n-1}n-n+1)(S_{2,n-1}n-S_{3,n-1}n-n+1)+2S_{2,n-1}{\left(S_{2,n-1}n-n+1\right)}^{2}}{2S_{1,n-1}^{4}n^{2}}$	0
*E_n_*, *T_k_*	$\frac{k^{2}{\left(k-1\right)}^{2}(n^{2}+2S_{1,n-1}n-9n+10)}{\left(n-1)(n-2\right)}$	$k^{2}{\left(k-1\right)}^{2}$
*E_n_*, *I_n_*	$\frac{2S_{1,n-1}^{3}n-S_{1,n-1}^{2}(6n-8)+S_{2,n-1}(n-1)(n-2)}{{\left(S_{1,n-1}-1\right)}^{4}(n-1)(n-2)}$	0
*B_n_*, *E_n_*	$\frac{4S_{1,n-1}n{\left(S_{2,n-1}n-n+1\right)}^{2}-2S_{2,n-1}^{2}(n^{2}+3n-6)n^{2}+S_{2,n-1}(3n-4)(n+6)n(n-1)+(n^{2}-10n+8){\left(n-1\right)}^{2}}{2n^{2}(n-1)(n-2)}$	$-\frac{\pi^{4}}{30}+\frac{\pi^{2}}{4}+\frac{1}{2}\approx 0.26159$
*I_n_*, *T_k_*	$\frac{k^{2}(k-1)[(k-1)S_{1,n-1}^{2}(n-1)(n-2)-2S_{1,n-1}(kn^{2}-4kn+n+2k)+(k-1)S_{2,n-1}(n-1)(n-2)+kn^{2}+n^{2}-9kn+3n+10k-6]}{\left(n-1)(n-2\right)}$	∞
*B_n_*, *I_n_*	$\frac{[S_{2,n-1}(n-1)(n-2)-4n+4S_{1,n-1}+4]{\left(S_{2,n-1}n-n+1\right)}^{2}}{{\left(S_{1,n-1}-1\right)}^{4}n^{2}(n-1)(n-2)}+\frac{2[S_{2,n-1}n-\left(S_{3,n-1}+1\right)(n-1)](S_{2,n-1}n-n+1)}{{\left(S_{1,n-1}-1\right)}^{3}n(n-1)}-\frac{2S_{2,n-1}^{2}n^{2}-S_{2,n-1}(3n-4)n-(n+4)(n-1)}{2{\left(S_{1,n-1}-1\right)}^{2}n^{2}}$	0
*B_n_*, *T_k_*	$\frac{k}{2}\left[\frac{\left[k{\left(k-1\right)}^{2}(3n+2)+4n](n-1\right)}{n^{2}}-(k+1)(k^{2}-3k+4)S_{2,n-1}\right]$	$\frac{1}{12}k[(18-\pi^{2})k^{3}-2(18-\pi^{2})k^{2}+(18-\pi^{2})k-4(\pi^{2}-6)]$

Expressions involving *E_n_* or *I_n_* apply for n≥3; expressions involving *B_n_* apply for n≥4. The value for (*H_n_*, *L_n_*) follows equation 18 of Arbisser *et al.* (2018). The expressions are obtained using equation 4 and Tables 2 and 3.

For pairs (*X_n_*, *Y_n_*), we simulate the values of $E\left[X_{n}/Y_{n}\right]$ and $\text{Var}\left[X_{n}/Y_{n}\right]$ under the coalescent model using ms (Hudson 2002), performing 100,000 replicate simulations for each tree size n=2,3,…,50. We plot the simulated values alongside the approximate values from Tables 4 and 5 in Figs. 2 and 4.

## Results

### Expectations of the ratios

The approximate expected values in Table 4, as approximations of ratios, have the form of rational functions. As *n* grows, the approximate expectations of $H_{n}/L_{n},\;H_{n}/I_{n},\;E_{n}/L_{n},\;E_{n}/I_{n},\;B_{n}/L_{n}$, and $B_{n}/I_{n}$ approach 0. This behavior is sensible when considering the properties of the coalescent model: in the numerators, *E_n_* has expectation 2 and $E\left[H_{n}\right]$ and $E\left[B_{n}\right]$ have bounded expectation in the limit as n→∞; in the denominators, *L_n_* and *I_n_* have expectations that grow without bound (Table 2). Similarly, approximate expectations of ratios $L_{n}/T_{k}$ or $I_{n}/T_{k}$ with *L_n_* and *I_n_* in the numerator and *T_k_* in the denominator grow to infinity as *n* increases. The approximation to $E\left[L_{n}/I_{n}\right]$ approaches 1 in the limit as n→∞: as the number of leaves in the tree grows, internal branches occupy an increasingly large fraction of the total branch length.

For pairs of variables that both have finite expectation, the approximate expectations of their associated ratios—$H_{n}/T_{k},\;E_{n}/H_{n},\;E_{n}/T_{k},\;B_{n}/H_{n},\;B_{n}/E_{n}$, and $B_{n}/T_{k}$—also approach finite values in the limit as n→∞. It is interesting to observe that although $\underset{n\to \infty }{\lim}E\left[E_{n}\right]=\underset{n\to \infty }{\lim}E\left[H_{n}\right]=2$ (Table 2), $\tilde{E}\left[E_{n}/H_{n}\right]=\pi^{2}/3-2\approx 1.28987\neq 1$. In other words, although expectations of the individual variables approach the same value, we expect $E_{n}/H_{n}$ to be somewhat larger than 1 on average.

For each of the 10 pairs of variables among $\left\{H_{n},L_{n},E_{n},I_{n},B_{n}\right\}$, the approximate expectations from Table 4 are plotted in Fig. 2 together with the simulated values. Although some divergences are present for small *n*, the approximate and simulated values match closely.

The approximate ratios involving *T_k_* are shown in Fig. 3 as functions of *k* for each of three values of *n*. *L_n_* is the fastest-growing variable according to the expression for its expectation (Table 2), and the graph for $\tilde{E}\left[L_{n}/T_{k}\right]$ is topmost in all three plots. As expectations of *H_n_* and *E_n_* are close (Table 2), the graphs for $\tilde{E}\left[H_{n}/T_{k}\right]$ and $\tilde{E}\left[E_{n}/T_{k}\right]$ are close in Fig. 3.

### Variances of the ratios

The limits of approximations of variances of ratios are presented in Table 5. They behave similarly to the expectations in Table 4. Because *L_n_* and *I_n_* have expectations that grow without bound, for ratios $H_{n}/L_{n},\;H_{n}/I_{n},\;E_{n}/L_{n},\;B_{n}/L_{n},\;E_{n}/I_{n},\;B_{n}/I_{n}$—with *L_n_* or *I_n_* in the denominator—the limits of the variance approximations are 0. As *n* grows, the denominators grow much faster than the numerators, and the values are therefore increasingly concentrated around 0. Hence, the variances also approach 0.

Because *L_n_* and *I_n_* are much larger than the coalescence times *T_k_*, approximations to variances of $L_{n}/T_{k}$ and $I_{n}/T_{k}$ diverge to infinity as *n* increases. Interestingly, however, the approximate variance of $L_{n}/I_{n}$, a ratio of two quantities with diverging expectations, approaches 0.

The variance approximations with finite nonzero limits are those for $H_{n}/T_{k},\;E_{n}/H_{n},\;E_{n}/T_{k},\;B_{n}/H_{n},\;B_{n}/E_{n}$, and $B_{n}/T_{k}$. All give ratios of two variables with finite expectation and variance as n→∞ (Table 2).

Figure 4 shows the expressions from Table 5 together with the simulated values. Compared to the plots of expectations of ratios (Fig. 2), differences between the simulated and approximate variances are prominent at small *n*. For the variances of $H_{n}/L_{n},\;B_{n}/H_{n}$, and $B_{n}/L_{n}$, the simulated and approximate values differ substantially even as *n* increases. Because the theoretical value of $\text{Cov}[E_{n},B_{n}]$ that contributes to the approximate variance of $B_{n}/E_{n}$ is itself an approximation, one of the larger differences between simulation and approximation occurs for the plot for $\tilde{\text{Var}}\left[B_{n}/E_{n}\right]$.

Figure 5 shows variances of ratios involving *T_k_* for varying *k*, for each of three values of *n*. Qualitatively, the values for approximate variances behave similarly to expectations in Fig. 3: in particular, the vertical placement of the curves follows the same order. Our approximations to the variances of $L_{n}/T_{k}$ and $I_{n}/T_{k}$ grow fastest, as the numerators are typically large and the expected value of the denominator *T_k_* decreases as *k* grows. Approximations to variances of $H_{n}/T_{k},\;E_{n}/T_{k}$, and $B_{n}/T_{k}$ all display much slower growth; for these quantities, the expectations of numerators of the ratios are bounded above by 2 for all *n*.

## Discussion

In this article, we have computed approximations to expected values and variances of ratios of various branch lengths under the standard coalescent model. We have considered all 15 possible pairs of variables in $\left\{H_{n},L_{n},E_{n},I_{n},B_{n},T_{k}\right\}$, a set of variables whose properties have been studied in detail individually. We have also assessed the accuracy of approximations to the expectation and variance by comparing them with values computed by simulation. We have observed that the approximate expressions behave in a way that matches mathematical intuition about the behavior of random variables associated with the branch lengths.

In plots of the various approximations, we have illustrated how the random variables relate to each other, both among $\left\{H_{n},L_{n},E_{n},I_{n},B_{n}\right\}$ (Figs. 2 and 4) as well as between pairs including one of $\left\{H_{n},L_{n},E_{n},I_{n},B_{n}\right\}$ along with *T_k_* (Figs. 3 and 5). As *n* grows large, the ratios involving *L_n_* and *I_n_* have nearly identical behavior in the plots, an observation that is explained by the fact that internal branches take up increasingly large fractions of the total branch length. In the limit as n→∞, expectations of both *H_n_* and *E_n_* approach a constant value of 2 (Table 2), and $\text{Cov}[H_{n},E_{n}]$ approaches 0 (Table 3). However, we observed that $\underset{n\to \infty }{\lim}\tilde{E}\left[E_{n}/H_{n}\right]=\pi^{2}/3-2\approx 1.28987$ is not equal to $\underset{n\to \infty }{\lim}E\left[E_{n}\right]/\underset{n\to \infty }{\lim}E\left[H_{n}\right]=1$. For the ratio $B_{n}/E_{n}$, the approximation aligns with the naive prediction, $\underset{n\to \infty }{\lim}\tilde{E}\left[B_{n}/E_{n}\right]=\pi^{2}/6-1=\underset{n\to \infty }{\lim}E\left[B_{n}\right]/\underset{n\to \infty }{\lim}E\left[E_{n}\right]$, even though $\text{Cov}[E_{n},B_{n}]$ is also zero in the limit (Table 2). For *B_n_* and *H_n_*, which possess a high correlation, $\underset{n\to \infty }{\lim}\tilde{E}\left[B_{n}/H_{n}\right]=\pi^{4}/18-\pi^{2}/6-\zeta (3)-2\approx 0.56463$, whereas $\underset{n\to \infty }{\lim}E\left[B_{n}\right]/\underset{n\to \infty }{\lim}E\left[H_{n}\right]=\pi^{2}/6-1\approx 0.64493$.

Previously, we evaluated covariances and correlation coefficients under the coalescent model for the pairs of variables that we consider here, obtaining exact covariances and correlations for 13 of 15 pairs and approximations for the other two. We obtained limiting expressions for these covariances and correlations as n→∞. The approximate values that we have provided here for expectations and variances of ratios make use of these previous results concerning covariances, adding to the understanding of the properties of joint distributions of pairs of genealogical variables in coalescent theory.

Many statistical tests of population-genetic models rely on a model prediction of an equivalence between two quantities, framed as a null hypothesis that a test statistic equals a particular value. The prediction is often formulated as a null hypothesis that a difference between two quantities equals 0 or that their ratio equals a null value such as 1. In coalescent theory, tests that evaluate site-frequency spectra for agreement with predictions of coalescent models tend to use differences or other linear combinations (Zeng *et al.* 2006; Achaz 2009; Ferretti *et al.* 2010, 2017; Ronen *et al.* 2013; Fu 2022). However, several modeling studies and inference procedures in coalescent theory do emphasize ratios (Slatkin 1996; Uyenoyama 1997; Schierup and Hein 2000; Rosenberg and Hirsh 2003; Eldon 2011; Arbisser *et al.* 2018), as do some test statistics (Schlötterer 2002; Lohse and Kelleher 2009). Widely used tests in the area of molecular evolution, such as tests of the relative count of nonsynonymous and synonymous substitutions and the McDonald–Kreitman test of polymorphism and divergence, also make use of ratios (Yang 2014).

The choice of a difference or a ratio in formulating a test statistic can rely on several factors. Ratios are unitless, so that their values do not depend on conventions chosen during computation (e.g. scaling time in units of *N* or 2*N*). Ratios might take values in a prescribed range that can be simply interpreted, such as the range of the coalescent ratio $H_{n}/L_{n}$ from $\frac{1}{n}$ to $\frac{1}{2}$ (Arbisser *et al.* 2018). However, the statistical properties of random variables formulated as differences are generally easier to compute from the properties of the separate random variables whose difference is taken than are the properties of corresponding statistics formulated as ratios. In general, corresponding differences and ratios in coalescent theory have not been formally compared for features such as their power to reject the null hypothesis when processes such as natural selection or population or species divergence affect the shapes of evolutionary trees. Our work to obtain approximate expectations and variances of ratios can augment understanding of scenarios in which coalescent ratios are considered, and it can assist in evaluating the relative utility of difference-based and ratio-based statistics.

We have found that approximations for fixed *n* and in the limit as n→∞ are quite accurate in predicting the expected values seen in coalescent simulations of the ratios (Fig. 2). For the variances, the approximations are generally less accurate, although in most cases, graphs of the approximations and simulated values have similar shape (Fig. 4). These approximations are obtained from a Taylor approximation for the variance of a ratio (equation 4), and higher-order approximations of this variance could potentially be applied by use of Taylor’s theorem; as the order of the approximation increases, however, the complexity of the resulting formula also increases. For those variances for which the approximation and simulation are not close in Fig. 4, we advise caution in using the variances in settings in which a precise approximation is needed.

## Data Availability

The ms command for simulations is ms *n* 100,000 -T, where *n* is taken from {2,3,…,50} and gives the number of leaves of simulated trees.
